# Combining motion performance with EEG for diagnosis of mild cognitive impairment: a new perspective

**DOI:** 10.3389/fnins.2024.1476730

**Published:** 2024-12-04

**Authors:** Xianglong Wan, Shulin Xing, Yifan Zhang, Dingna Duan, Tiange Liu, Danyang Li, Hao Yu, Dong Wen

**Affiliations:** ^1^School of Intelligence Science and Technology, University of Science and Technology Beijing, Beijing, China; ^2^Key Laboratory of Perception and Control of Intelligent Bionic Unmanned Systems, Ministry of Education, Institute of Artificial Intelligence, University of Science and Technology Beijing, Beijing, China; ^3^Sports Department, University of Science and Technology Beijing, Beijing, China

**Keywords:** mild cognitive impairment (MCI), brain computer interface (BCI), motion performance, diagnosis efficiency, combination

## 1 Introduction

The prevalence of mild cognitive impairment (MCI) is rapidly increasing with the growing global elderly population. Approximately 15% to 20% of individuals aged 65 and above suffer from MCI (Petersen et al., 2018). Nowadays, the Mini-Mental State Examination (MMSE) and the Montreal Cognitive Assessment (MoCA) are commonly used scales for MCI detection (Nasreddine et al., 2005; Ciesielska et al., 2016). Except for the scales, the fluid biomarkers, such as plasma phospho-tau217 and total-tau in cerebrospinal fluid, can also be used for MCI detection (Olsson et al., 2016; Palmqvist et al., 2020; Moscoso et al., 2021). Even though the diagnosis of MCI based on these biomarkers demonstrates outstanding accuracy and stability, it is not without its limitations. Lumbar puncture stands as a frequently conducted invasive procedure in clinical practice, of cerebrospinal fluid samples (Doherty and Forbes, 2014). It requires a relatively long postoperative recovery time and may lead to complications, such as headache, cranial nerve palsy, and reversible tonsillar descent (Evans, 1998).

Although the main symptom of MCI is memory impairment, motor dysfunction has been previously described as a common characteristic in the patients with cognitive impairments (Montero-Odasso et al., 2014). In comparison to normal subjects, individuals with MCI exhibit differences in some motions, such as walking and balance. According to Shin et al. (2011) and Rosenberg et al. (2023), motion performance indices extracted from them can be used for diagnosing MCI. However, as the physical activity is only weakly associated with the global cognition (Iso-Markku et al., 2024), motion performance tests could not be used to directly detect subtle alterations in the cognitive functions of MCI patients. To address the aforementioned issues, it is imperative to discover a novel approach to enhance both diagnostic efficiency and accuracy.

We postulate on the combination of EEG and motion performance tests as a crucial tool for MCI diagnosis. The electroencephalogram (EEG) is a non-invasive electrophysiological monitoring technique used to monitor brain electrical activity, offering superior temporal resolution. Owing to distinctions in EEG features between individuals with MCI and the normal ones, such as power spectral density (PSD) and band power, EEG can be independently utilized in the diagnosis of MCI (Meghdadi et al., 2021; Mitsukura et al., 2022). The application of automated feature learning techniques (such as deep learning and machine learning) in MCI diagnosis enables EEG-based diagnostic methods to achieve high accuracy and AUC (Al-Nuaimi et al., 2018, 2021; Meghdadi et al., 2021). Therefore, combining motion with EEG for MCI diagnosis can compensate for the lack of accuracy and AUC when using motion alone to diagnose MCI (You et al., 2020). Moreover, although MCI patients can maintain relatively independent activities in daily life, they are often affected to varying degrees when performing tasks with high cognitive load (Jekel et al., 2015; Klotzbier and Schott, 2017). Mainstream EEG-based MCI diagnostics typically use resting-state EEG, which makes it difficult to assess which daily activities are affected in MCI patients. This study comprehensively reviews the literature on the MCI diagnosis with EEG and motion performance tests. In consideration of prior studies, we additionally delve into the combination of EEG and motion performance indices for diagnostic purposes, and propose our own opinions about the development trends of this diagnostic strategy. Finally, we propose recommendations to advance research in the diagnosis of MCI.

## 2 MCI diagnosis with motion performance

Motion is governed by the brain and spinal cord nerves. The connection between motion, cognition, and the nervous system is closely intertwined (Gentsch et al., 2016). Extensive study indicates that many non-cognitive symptoms, such as motor function loss, major depression and disruptive behaviors, are associated with neurodegenerative diseases (Goldman et al., 1999; Lopez et al., 2005; Haaxma et al., 2010; Buchman and Bennett, 2011).

In recent years, a plethora of study findings has shown that gait dysfunction is a prevalent characteristic among patients with MCI. As gait plays a crucial role in discriminating pathology and identifying the progression of the disease (Morris et al., 2016), gait parameter can serve as one of the motion performance indices for diagnosing MCI (Montero-Odasso et al., 2014). In this study, an electronic walkway was utilized to assess the gait performance of individuals with MCI as well as that of normal individuals. The participants carried out dual-tasks walking, and the coefficient of variation for stride time was recorded, respectively. It is a motor-divided attention task that requires individuals to walk while doing a cognitively demanding task (reciting words or calculations). The results indicated deficits in gait speed and stride time variability for MCI patients, demonstrating the feasibility of gait as a diagnostic tool for potential MCI patients. Moreover, the differences in gait patterns between patients with MCI and normal individuals can be validated through the trail walking test (TWT) under different cognitive loads, and become more pronounced as the cognitive load increases during walking (Klotzbier and Schott, 2017). Therefore, the diagnosis of MCI in elderly adults can be achieved through the evaluation of completion time and errors as long as the TWT is sufficiently sensitive. The development of technologies such as machine learning allows researchers to process these movement data more quickly and effectively. Based on a machine learning model, researchers diagnose MCI by analyzing only a few seconds of computer mouse movement data, with the model achieving an average accuracy of 79.8% (Hanczár et al., 2022).

Furthermore, static postural balance during standing is another motor function that is critical to quality of life and seems to have a direct association with cognitive function (Tell et al., 1998). In the eyes-open condition, there is no significant difference in balance function between MCI patients and the normal control group (Leandri et al., 2009). However, in the eyes-closed condition, MCI patients exhibit early changes in balance function, particularly in the parameters of anterior-posterior sway. Moreover, the study results indicate a trend of increasing dependence vision among normal individuals, aMCI and AD patients. This implies that MCI-related balance deficits are related to impaired central processing of visual information.

## 3 MCI diagnosis with resting-state EEG

Quantitative analysis of EEG rhythms is a low-cost and potentially useful neurophysiological approach to the study of normal aging and dementia. This type of analysis includes the estimation of the power density of selected resting-state EEG frequency bands (Moretti et al., 2004; Jiang, 2005). Growing evidence indicates that the resting-state EEG can be utilized for detecting early abnormalities in neuronal function (Schaul, 1998; Musha et al., 2013). Hence, there is potential to extract biomarkers from the resting-state EEG of individuals with MCI for the purpose of early diagnosis. Compared to normal subjects, MCI patients are known to have differences in EEG frequency bands, namely delta (0.5−4 Hz) and theta (4−8 Hz) power increase, and alpha (8−13 Hz) power decrease (Moretti et al., 2004). Based on these differences, features extracted from EEG frequency bands have been utilized in diagnosing MCI and have enhanced diagnostic performance (Al-Nuaimi et al., 2021, 2018; Besthorn et al., 1997).

It is noteworthy that when comparing MCI with different neurodegenerative diseases, biomarkers derived from resting-state EEG may lack specificity. A study assessed the five traditional EEG frequency bands of 38 patients with neurodegenerative diseases in the resting state condition (Fonseca et al., 2013). The findings of EEG in Parkinson's disease dementia (PDD), Parkinson's disease (PD), and MCI demonstrate similar tendency in the changes of delta and theta frequency bands. Furthermore, another study with individual estimation of EEG frequency indicates an increase in alpha band power among MCI patients, contrary to the aforementioned conclusion (Meghdadi et al., 2021).

Complex network analysis treats brain networks as intricate network structures and investigates the interactive relationship between groups of neurons or across different regions of the brain (Bassett and Bullmore, 2009; Sporns, 2013). An important finding in EEG analysis of cognitive impairment is the decreased synchronization between brain regions (Azami et al., 2023). The power ratio between different frequency bands is also a biomarker for distinguishing between MCI and Healthy Controls (HC). Compared to the healthy control group, patients with Mild Cognitive Impairment (MCI) show a significantly lower beta/theta power ratio in the occipital region.

## 4 MCI diagnosis with the combination of motion performance and EEG

Cognitively demanding tasks such as dual-task walking aids in isolating the cognitive control component of locomotion, offering valuable information into the detection of cognitive decline (Hausdorff et al., 2008; Montero-Odasso et al., 2014). According to the above-mentioned limitations when using resting-state EEG alone, we believe that the combination of EEG and motion has resulted in higher diagnostic accuracy compared to using a single data source for classification. For example, a study revealed the capability of combining EEG data with gait kinematic parameters to differentiate individuals with MCI from those with normal cognitive function (You et al., 2020). The experiment was designed with two sequential steps to enhance the speed and accuracy of classification by concurrently utilizing gait and EEG data. First, healthy controls (HC) and patients were categorized based on gait data, and then directly extracting spatial and temporal features from original EEG data for distinguishing between MCI and AD patients. The findings suggested that the classification accuracy based on the fusing features from EEG and gait in the three-way classification of HC, MCI, and AD reaches 91.07%. The value is much higher than the method using one modal, because the classification accuracy when using gait data alone is 68.18%. Individuals with high risk of cognitive impairment and low risk of cognitive impairment may exhibit different underlying neural signatures while performing walking tasks under visual interference (De Sanctis et al., 2023). Specifically, Individuals at higher risk for cognitive impairment amplified theta localized to frontomedial and right central gyrus. In contrast, the brain response in lower risk individuals was specific to visually perturbed input and characterized by left central beta suppression. Furthermore, higher theta power was related to lower scores on the MoCA and stronger beta power suppression was related to higher scores on the MoCA. Another study combined gait with EEG has also demonstrated that multimodal signals combining EEG with gait kinematic parameters improved the ability to discriminate MCI individuals from normal controls (Min et al., 2022).

Compared to resting-state EEG, utilizing EEG collected during motion performance tests requires addressing the challenge caused by artifacts. To minimize artifacts interference, motion performance tests that allow concurrent EEG recording often impose restrictions on participants' movement amplitudes compared to traditional motion performance tests. For example, simple finger-tapping test (FTT) performed with a keyboard (Sharma et al., 2021). In this study, all subjects tapped the space-bar key for 10 s continuously until they were interrupted, while using the device (SOMNOscreen EEG 32) to collect data during this period. A total of 16 MCI patients aged 40 and above participated in the experiment. The results indicate that the FFT event provided the highest scores of classification, 91.23% accuracy and 92.38% specificity (the results under resting conditions show 87.22% accuracy and 79.49% specificity).

EEG collected during motion performance tests is susceptible to artifacts. Both physiologic and nonphysiologic sources of artifact may act as source of confusion with abnormality and lead to misinterpretation (Tatum et al., 2011). We advocate for researchers to continue improving the performance of movement-induced EEG artifacts removal methods. For example, in visual stimulation task, electrooculography (EOG) can be recorded and adaptive filtering can be employed to generate estimations of signals associated with eye movement artifacts. Subtraction of EEG recordings from these estimated signals helps to reduce the impact of artifacts. Alternatively, the head movements can be detected and quantified through sensor data analysis. The motion information can be correlated with EEG data collected during the same time period to identify and correct artifacts caused by head movements.

## 5 Discussion

As previously mentioned, research methods in this field are primarily classified into two types: one focuses on the combination of motion performance indices with resting-state EEG, while the another focuses on the analysis of EEG signals during motion performance tests. In future research, we suggest that researchers may attempt more diverse combinations. For example, researchers can collect EEG data from subjects during motion performance tests as well as resting-state EEG data before and after the tests, and analyze them with the kinematic characteristics of the MCI patients during the tests.

As previously mentioned, dual-task tests effectively links physical activity capacity with cognitive ability, offering more valuable information for diagnosing MCI patients. Due to the typically higher cognitive load experienced by humans during dual-task testing compared to single-task testing, we posit that the assessment of cognitive fatigue in MCI patients under dual-task conditions could be a promising diagnostic target. Cognitive fatigue is a psychobiological state with subjective, behavioral, and physiological consequences for an individual, such as heightened feelings of tiredness, increased reaction physical energy (D́ıaz-Garćıa et al., 2021). The symptoms of cognitive fatigue include obvious changes in EEG signals (Li et al., 2020). Under the same cognitive loads, MCI patients endure more cognitive fatigue compared to the normal individuals (Kukla et al., 2022; Zhang et al., 2023). According to our investigation, cognitive fatigue has been barely considered as one of the diagnostic criteria in the field of MCI diagnosis. In future research, we can analyze the EEG data of patients in dual-task tests to calculate the level of cognitive fatigue as an auxiliary diagnostic approach.

Disturbed interactions among brain regions have been shown to be associated with virtually all brain and mental disorders, as well as with brain injury and recovery (Sporns, 2013; San-Martin et al., 2021; Wen et al., 2019). From the previous discussion, it is evident that analysis of the EEG data for MCI patients under the dual-task tests will offer identifying diagnostic targets for MCI. In future research, we suggest researchers explore the interactions between brain regions of subjects during motion performance tests to achieve more accurate diagnosis and personalized treatment strategies. The diagnostic approach combining motion performance indices with EEG holds immense potential, yet it still faces several challenges. With ongoing in-depth study in this direction, the optimal solution for diagnosing MCI patients may be discovered in the future.
